# The effect of research on life satisfaction in middle-aged and older adults: physical disability and physical activity as a parallel and serial mediation analysis

**DOI:** 10.1186/s12877-023-03873-7

**Published:** 2023-03-27

**Authors:** Pei-Shan Li, Chia-Jung Hsieh, Ya-Ling Shih, Ya-Ting Lin, Chieh-Yu Liu

**Affiliations:** 1 School of Nursing, College of Nursing, National Taipei University of Nursing and Health Sciences, Taipei, 112 Taiwan R.O.C.; 2 Department of Nursing, Taipei Veteran General Hospital, Taipei, 112 Taiwan R.O.C.; 3 Department of Nursing, Yuanpei University of Medical Technology, Hsinchu, 300 Taiwan R.O.C.; 4 Department of Nursing, St. Mary’s Junior College of Medicine, Nursing, and Management, Yilan County, 266 Taiwan R.O.C.; 5 Department of Health Care Management, National Taipei University of Nursing and Health Sciences, Taipei, 112 Taiwan R.O.C.

**Keywords:** Frailty status, Physical disability, Physical activity, Life satisfaction, Older adults

## Abstract

**Background and objective:**

Maintaining the life satisfaction of frail middle-aged and older adults when they experience physical disability, lower activity status, or complex conditions that are related to each other is now an urgent issue. Therefore, the purpose of this study was to provide evidence for the impact of frailty in middle-aged and older adults on life satisfaction under the simultaneous occurrence and correlation of physical disability and physical activity status.

**Methods:**

Data from the 2015 Taiwan Longitudinal Study in Ageing (TLSA) were analyzed by PROCESS in SPSS to explore three different mediation models (N = 4,421). The first was a parallel mediation model for exploring life satisfaction in middle-aged and older adults with frailty through physical disability or physical activity. The second was a serial mediation model for examining physical disability and physical activity in causal chains linked with a specific direction of flow and to test all combinations. The third was a moderated mediation model for testing whether the indirect effect of frailty status on life satisfaction through physical disability or physical activity was moderated by age stratification.

**Results:**

Physical disability and physical activity partially mediated the relationship between frailty status and life satisfaction (IE_OVERALL_ = -0.196, 95% CI: -0.255 to -0.139). The causal path with the highest indirect effect was found to be that between frailty and physical disability; increased frailty led to higher physical disability, which in turn affected physical activity, leading to lower life satisfaction (IE = 0.013, 95% CI: 0.008 to 0.019). The different stratifications by age significantly increased the mediating effect of physical activity (Index of Moderated Mediation = -0.107, SE = 0.052, 95% CI: -0.208 to -0.005) but did not reduce the mediating effect of physical disability.

**Conclusion:**

This study provides evidence that physical activity and physical disability influence the development of frailty. It also has a significant impact on the life satisfaction of middle-aged and older adults.

**Supplementary Information:**

The online version contains supplementary material available at 10.1186/s12877-023-03873-7.

## Introduction

As the global population structure trends towards ageing, it can be expected that the life expectancy of older people will continue to increase, which in turn will cause the public to change the model and concept of elderly care [1]. Frailty status (FS) is common in older people. The morbidity rate in the frail stage was 12–24%, and the morbidity rate in the pre-frail stage was as high as 46–49% [2]. When older adults respond to the onset of ageing, they begin to feel systemic skeletal muscle loss, muscle strength decline, and organ system degeneration[3]. This progressively reduces the physical activities that could otherwise be mastered, gradually limiting mobility [4]. This allows ageing to form the basis for the FS of older adults [5]. Frailty not only affects the physiological health of older adults but also causes a decline in physical activity (PA) functions, and such a decline will affect their daily lives, leading to a decline in life satisfaction (LS) [6]. The long-term predicament of frailty issues highlights the importance of achieving healthy sustainability in elderly care goals in order to maintain LS.

Many older adults are often burdened by different states such as ageing, frailty, or disability, but ageing and frailty or disability are often compared due to their similarities [7]. When older adults begin to have difficulty performing basic activities of daily living, which marks the progression toward physical disability (PD) [8]. Further study results show that nearly 60% of older adults have lost the ability to live independently due to PD [9]. The evidence suggests that prolonged periods of low PA levels in older adults are highly related to frailty morbidity rates and increased FS [10]. The World Health Organization (WHO) recommends that older adults engage in physical activity with a variety of components on three or more days a week to enhance functional capacity [11]. Thus, increasing the level of PA in daily life will help older adults control the degree of FS and the risk of frailty [12]. Most studies on frail older adults in the community have shown that FS is related to limited PA function [13]. Further studies have set PA as a predictor of frailty in older adults and emphasised the importance of PA in maintaining health in old age and promoting healthy ageing [14]. Accordingly, we speculated that PD and PA may mediate the direct and indirect relationships between LS and FS. At present, the evidence on the effects of reducing the development of PD, increasing the level of PA, or improving LS in older adults is relatively fragmented. The factors that affect LS among older adults have been extensively studied and found to include demographic, physiological, and mental health-related factors [15]. For example, age and gender differences, as well as chronic conditions and health restrictions among middle-aged and older people, were found to increase the risk of LS [16, 17]. Current avenues of study are based on the consideration that the LS of older people will be affected by health-related conditions [18]. When older adults are in a frail state, it is still controversial whether to directly assess the impact of PD or PA [3]. It can be seen that the levels of PD and PA of frail older adults are serious threats to LS [6]. Due to a lack of evidence, the mechanisms underlying the mediation effects of PD and PA were not considered. Accordingly, it is imperative to confirm the association between FS and LS by utilizing a database and to determine how to improve LS in middle-aged and older adults.

While there is a correlation between PA and the morbidity rate of PD [19], there is currently a lack of evidence to explore the impact of PD on LS in frail middle-aged and older adults, and past literature has not validated the relationship between PA and LS. Maintaining LS has become an urgent issue for the frail middle-aged and elderly populations who experience PD, PA status or interrelated complex situations. Following the above inference, it is crucial to identify PD and PA as potential explanations for the association between FS and decreased LS. Therefore, one hypothesis of this study was that PD and PA would be mediating factors between FS and LS in middle-aged and older adults. The second research hypothesis was that the mediation effect would be different between middle-aged and older individuals. The purpose of the research was to provide evidence for the simultaneous relationship, interrelationship, and causality between PD and PA in middle-aged and older adults and their impact on LS.

## Methods

### Data collection

This study was an observational and retrospective study based on the “Taiwan Longitudinal Study in Ageing (TLSA)”. The TLSA was conducted by the Bureau of Health Promotion under Taiwan’s Department of Health and established in response to the ageing of Taiwan’s population and policy need. The development of the TLSA database was a collaborative effort of the Taiwan Health Promotion Bureau, the University of Michigan’s Population Research Center, and the University of Michigan’s Institute of Gerontology to jointly conduct cohort surveys, data collection, and follow-up on the physiological, psychological, and social status of the middle-aged and older-age populations in Taiwan. The TLSA subjects were selected based on a three-stage probability and stratified random sampling method to select middle-aged and older samples representative of Taiwan. The database maintained quality through a structured questionnaire, which was validated by experts. All data in the database are collected with strict process control. The first wave of TLSA surveys began in 1989 and recruited subjects over the age of 60. The second wave of TLSA surveys began in 1996 and recruited subjects over the age of 50 [20]. TLSA data have previously been used in gerontological studies related to health in middle-aged and older adults [21–25].

### Participants

This study sampled data added to the TLSA database in 2015 for analysis. The 8th wave of the TLSA targeted residents with households registered in Taiwan at the end of April 2015. The TLSA database was collected through individual interviews with participants who completed the training. The structured questionnaire was validated by experts and gathered data on characteristics, health status, social support, employment status, leisure and social participation, aging mentality, economic status, etc. The newly added sample included 5,304 middle-aged and older adult respondents aged 50 years or older at the end of April 2015. The exclusion criteria were as follows: (i) cognitive impairment with a score of less than 2 on the Short Portable Mental State Questionnaire (SPMSQ); 324 participants were excluded based on an SPMSQ score of less than 2. (ii) missing values in the variables; a total of 559 participants with missing values in the variables were excluded. After screening by inclusion and exclusion criteria, a total of 4,421 middle-aged and older adults were included in this study.

### Measures

#### Frailty status as predictors variables

FS was defined according to the Fried criteria, and characteristics included shrinking, exhaustion, slowness, weakness, and low PA. The frailty score of 0 to 5 points was based on five characteristics, and a higher score indicated greater severity of FS. Additionally, no characteristics indicated the non-frailty stage; less than 2, the pre-frail stage; and over 3, the frail stage [26]. The Fried criteria have high validity and reliability and are the most commonly used measure of frailty [27]. In the TLSA questionnaire, no widely accepted operational definition of frailty was available. Based on the Survey of Health, Ageing and Retirement in Europe (SHARE), modifications have been made to the definition of frailty to fit the data [23]. Subjects who reported frequent loss of appetite in the previous week were defined as “shrinking“[21, 22]. Subjects who answered “I could not get going” or “I felt everything I did was an effort” frequently or for most of the previous week on the Center for Epidemiologic Studies Depression Scale (CES-D) were defined as “exhaustion” [21, 22]. Subjects who were unable to walk a distance of 200 to 300 m or found it difficult were defined as “slowness” [21, 22]. Those who found it difficult to carry 12 kg of groceries were defined as “weakness” [21, 22]. Those who did not partake in walking, hiking, jogging, gardening, or other outdoor activities at least once or twice a week were defined as “low PA” [21, 22]. Cronbach’s alpha for the FS in this study was 0.809, demonstrating good internal consistency.

#### Physical disability as mediating variables

The definition of PD included activities of daily living (ADL), instrumental activities of daily living (IADL), and strength and mobility. The Chinese versions of the ADL and IADL measures have been reported to have established validity and reliability [28, 29]. The range was from 0 to 17, with a higher score indicating more severe PD. Based on the published literature, we selected suitable replacement items from the original TLSA questionnaire to fit the data[20]. ADLs include bathing, dressing and undressing, eating, going to the toilet, moving in and out of bed, walking, and going to the toilet [24]. IADLs include buying personal supplies, traveling by car or by train, performing light housework, and dialing phone numbers [24]. Measurements of strength and mobility were used to identify disability in physical function, including standing for 15 min, squatting down, raising hands over head, carrying a load of 20 pounds, running 20–30 m; walking 200–300 m, and climbing a flight or two of stairs [24]. Cronbach’s alpha for the PD in this study was 0.897, demonstrating good internal consistency.

#### Physical activity as mediating variables

The PA score was based on the dose (> 30 min/day), frequency (days per week), and intensity (causing sweating or not) of exercise. The scores indicated three levels, with a higher level indicating more PA [30]. Based on the literature, the PA items from the original TLSA questionnaire were used to calculate the score [25]. Subjects who reported that they did no exercise were defined as “no PA”, and 1 point was given [25, 30]. Subjects who reported exercising 1 to 2 times a week were defined as “moderate PA” and 2 points were given [25, 30]. Those who reported exercising more than 3 times per week and for at least 30 min per session were defined as “high PA” and 3 points were given [25, 30]. On the one hand, no PA and moderate PA were investigated. On the other hand, the levels of physical activity were high enough to analyze whether the older adults met the WHO-specified level of PA. Cronbach’s alpha for the PA score in this study was 0.871, demonstrating good internal consistency.

#### Middle-aged and older as moderation variables

The stratification by age was based on the definition of the World Health Organization [31], and the population structure of older people in Asia [32], which sets 60 years old as the standard for middle-aged (50–59 years old) and older (≥ 60 years old).

#### Life satisfaction as an outcome variable

The items and dimensions on the short-form Life Satisfaction Index (LSI-SF) are based on the theory of LISA. The three dimensions are “Zest vs. Apathy,“ “Resolution and Fortitude,“ and “Congruence Between Desired and Achieved Goals.“ A total of 6 items are listed, and only item 2 is reverse coded. It is designed with dichotomies for each item, with scores ranging from 0 to 6 points. A higher score indicates higher LS. The LSI-SF is valid and reliable for measuring LS in older people. The internal consistency reliability of Cronbach’s alpha coefficients was 0.81. Its validity has been supported by findings of sufficient convergent and discriminant validity [33].

### Statistical analysis

In this study, big data analytics were used to test PD and PA as mediating variables in the relationship between FS and LS. First, descriptive statistical analysis was performed in SPSS for Windows (version 22.0; SPSS Inc., Chicago, IL, USA). The numbers, percentages, means, and standard deviations of the sociodemographic characteristics of the subjects and various variables are shown. Correlation analysis of variables using univariate linear regression was used to examine the associations among FS, PD, PA, and LS. The mediation analyses were performed in the PROCESS macro for SPSS developed by Hayes, using models 4, 6, and 15 to explore three different mediation patterns [34]. Model 4 (model as a parameter) in the PROCESS function was used for the parallel mediation model to explore how FS in middle-aged and older adults affects LS through PD and PA. In the current study, model 4 was chosen to test the mediating effect of FS on LS via PD or PA. Model 6 for the serial mediation model was used to explore three different relationships through the two mediators of PD and PA. In the current study, model 6 was chosen to test whether age stratification moderates the indirect effects of FS on LS via PD or PA. Model 15 (moderated mediation) for the moderated mediation model was used to explore the effects of frailty in PD and PA on LS that were moderated by age stratification. In the current study, model 15 was chosen to test the mediation of PD and PA in causal chains linked to a specific direction of flow and to test all combinations. In models 4, 6, and 15, we adjusted for age, gender, and the number of chronic diseases to account for carryover effects of LS [16, 17]. This study followed the suggestion by Hayes and Preacher to conduct analyses of different paths for independent variables. The lower limit (LL) to the upper limit (UL) in the 95% confidence intervals (CIs) for the indirect effect (IE) did not include zero, indicating that the mediation was significant.

## Results

### Participant characteristics

The study subjects included 2,155 males and 2,266 females. The majority of the subjects were older (58.20%), had completed primary school (43.3%), and lived with a spouse (73.9%). There were significant differences in age, gender, education, and marital status among subjects in the non-frailty, pre-frailty, and frailty stages. In addition, the subjects in the frail stage had higher scores in PD (8.30 ± 4.55), largely had no PA (3.9%), and had significantly lower LS (3.57 ± 1.79) (shown in Table 1). FS was positively correlated with PD (r = 0.689, p < 0.01) but negatively correlated with PA (r =- 0.367, p < 0.01). LS was negatively correlated with FS (r = -0.248, p < 0.01) and PD (r = -0.215, p < 0.01).

**Table 1:** Comparison of participant characteristics between non-frail groups, pre-frail groups, and frail groups

Variables	Total (n = 4421)		Non-frail stage (n = 2018)		Pre-frail stage (n = 2182)		Frail stage (n = 221)		
	N (Mean)	% (SD)	N (Mean)	% (SD)	N (Mean)	% (SD)	N (Mean)	% (SD)	p / F
Age									< 0.001
Middle-aged	1849	41.80	842	18.60	994	22.60	28	0.60	
Older	2572	58.20	1194	27.00	1185	26.80	193	4.40	
Gender									< 0.001
Male	2155	48.70	1041	23.50	1048	23.70	66	1.50	
Female	2266	51.30	977	22.10	1134	25.70	155	3.50	
Education level									< 0.001
Primary school	1913	43.30	765	17.30	989	22.40	159	3.60	
Middle school	788	17.80	340	7.70	422	9.50	26	0.60	
High school	1239	28.00	647	14.60	562	12.70	30	0.70	
University	481	10.90	266	6.00	209	4.70	6	0.10	
Spousal status									< 0.001
With a spouse	3268	73.90	1583	35.80	1582	35.80	103	2.30	
Without a spouse	1153	26.10	435	9.80	600	13.60	118	2.70	
PA									< 0.001
No	1640	37.10	376	8.50	1090	24.70	174	3.90	
Moderate	668	15.10	300	6.80	347	7.80	21	0.50	
Highly	2113	47.80	1342	30.40	745	16.90	26	0.60	
FS (0–5)	(0.74)	(0.87)	2018 (0.00)	45.60 (0.00)	2182(1.15)	49.40 (0.36)	221(3.35)	5.00 (0.61)	< 0.001
PD (0–17)	(1.11)	(2.49)	(0.24)	(0.69)	(1.19)	(2.03)	(8.30)	(4.55)	< 0.001
LS (0–6)	(4.81)	(1.49)	(5.09)	(1.32)	(4.67)	(1.53)	(3.57)	(1.79)	< 0.001

FS: frailty status; PA: physical activity; PD: physical disability; LS: life satisfaction

### Parallel mediation model

**Fig. 1:**
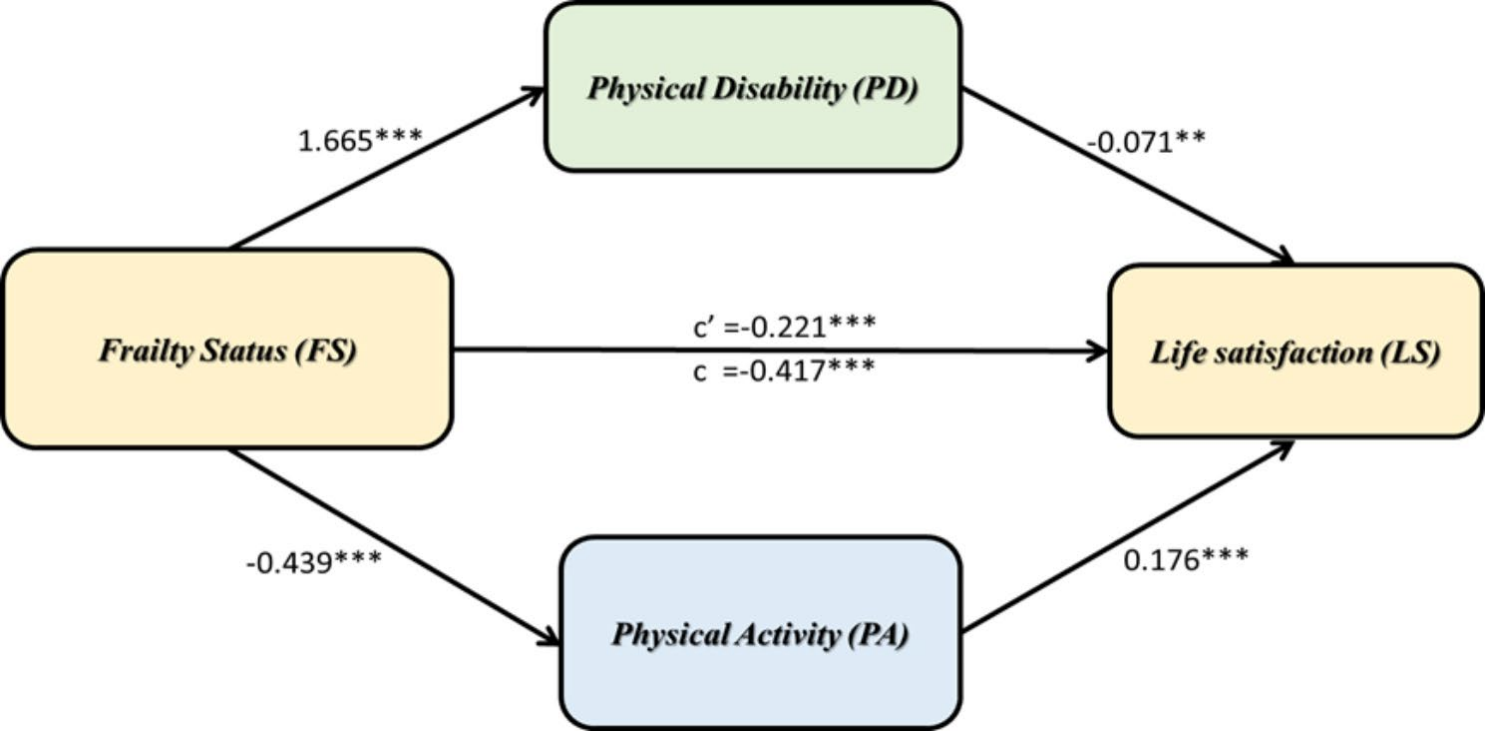
Parallel mediation model (n = 4,421) Standardised effects are presented. The Parallel mediation model was adjusted for age, gender, and the number of chronic diseases. Indirect effects of frailty status on life satisfaction through physical disability and physical activity. The effects on the direct path from frailty status to life satisfaction depict the direct effect and the total effect. **p < 0.01, ***p < 0.001

A parallel mediation model was to explore the FS of middle-aged and older adults in LS through PD or PA. After adjustments for age, gender, and number of chronic diseases, the results showed that the effect of FS was negatively correlated with LS (β_total_ = -0.417, SE = 0.026, p < 0.001). PD and PA had a mediating effect on FS with LS (shown in Fig. 1). Overall, PD and PA partially mediated the relationship between FS and LS (IE_OVERALL_ = -0.196, 95% CI: LL = -0.255 to UL = -0.139, indicating that middle-aged and older adults with increased FS may have higher levels of PD and lower levels of PA. Middle-aged and older adults with lower levels of PD and reduced levels of PA may have lower LS. The two mediating variables, PD and PA, were found to significantly contribute to the overall indirect effects. Specifically, there was a statistically significant indirect effect of FS on LS through PD (IE_PD_ = -0.119, 95% CI: LL = -0.170 to UL = -0.068). Therefore, middle-aged and older adults who experienced increased FS were more likely to feel PD, and through high levels of PD, they were more likely to report lower LS. In addition to this, there was a statistically significant indirect effect of FS on LS through PA (IE_PA_ = -0.077, 95% CI: LL = -0.101 to UL = -0.055). This showed that middle-aged and older adults who experienced increased FS were more likely to feel lower PA, and through reduced levels of PA, they were more likely to report lower LS.

### Serial mediation model

**Fig. 2:**
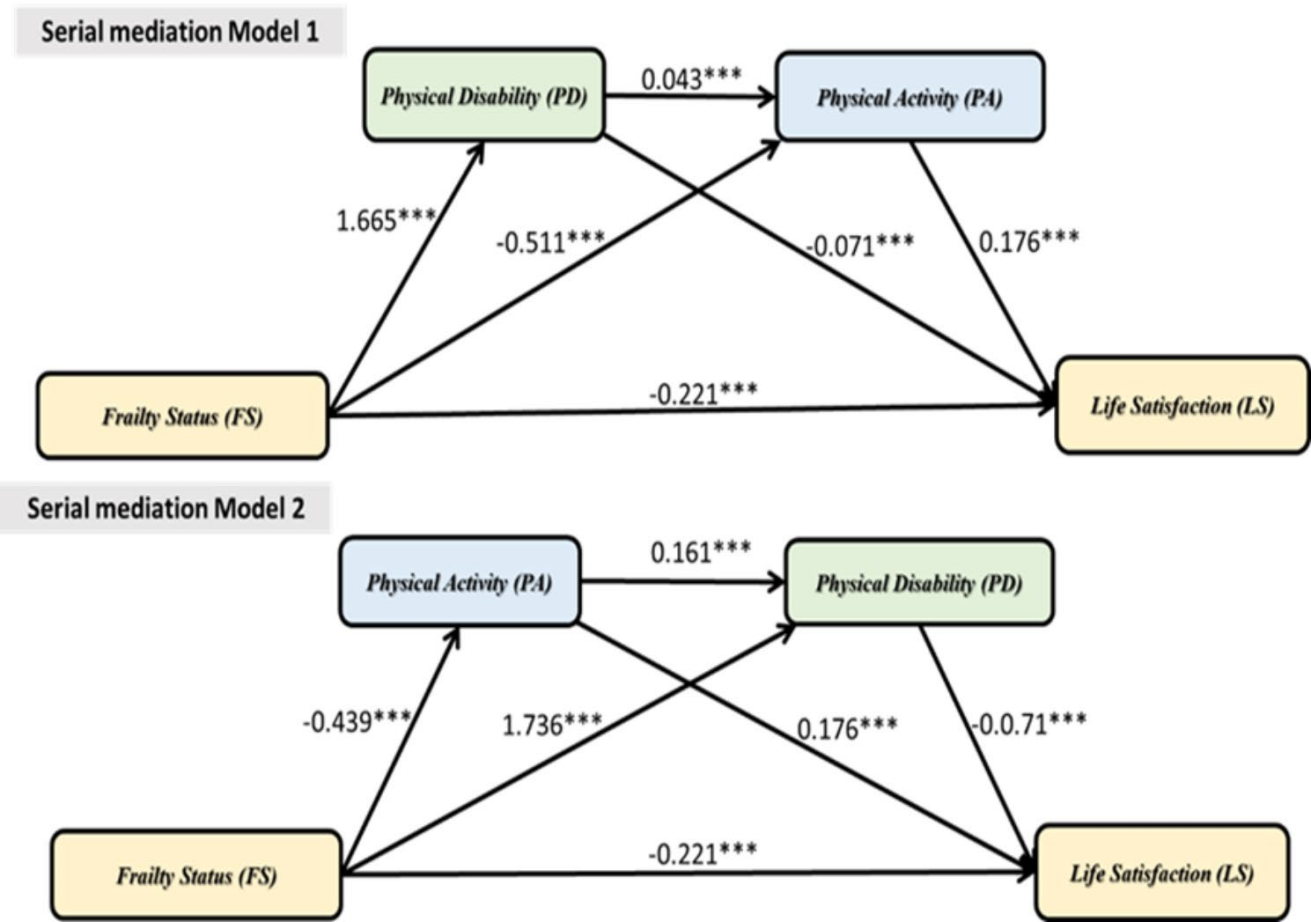
Serial mediation model (n = 4,421) Evaluating physical disability and physical activity as mediators of the relationship between frailty status and life satisfaction, respectively. The serial mediation model was adjusted for age, gender, and the number of chronic diseases. The graph illustrates the effects of the direct paths linking frailty status to each mediator and among mediators resulting from the serial mediation model, in which all the direct and indirect effects are statistically significant. ***P < 0.001

A serial mediation model was to examine the mediation of PD and PA in causal chains linked with a specific direction of flow and to test all combinations. After adjustments for age, gender, and the number of chronic diseases, the results showed that PD and PA in serial causal order mediated the relationship between FS and LS, and the ratio of the overall indirect effects to the total effect was 0.417 (95% CI: LL = -0.469 to UL = -0.365). The total indirect effects and the ratio of the total effect were the same as in the parallel mediation model described earlier. The positive and negative effects indicated the severity of FS, which led to increased PD and a decrease in PA. According to the results of Serial Mediation Model 1, the more severe the FS was, the more it would contribute to PD. A higher PD resulted in lower levels of PA, which in turn contributed to lower LS. According to the results of Serial Mediation Model 2, the more severe the FS was, the more it would contribute to PA. The lower levels of PA resulted in higher levels of PD, which in turn contributed to lower LS (shown in Fig. 2).

Since two mediators of PD and PA were used, two different causal order models were produced. The two models were compared based on the significant paths created by each different causal order of the mediators. Serial Mediation Model 1 and Serial Mediation Model 2 yielded three significant paths, respectively. A total of six paths were statistically significant. Additionally, Serial Mediation Model 1 and Serial Mediation Model 2 yielded a significant indirect path involving both PD and PA as mediators in a causal chain, respectively (Path C). The indirect paths involving PD or PA, one after the other and vice versa, were statistically significant in all the serial mediation models. Path C in Serial Mediation Model 1 (FS → PD → PA → LS) had the highest ratio of indirect to total effect of all the models (IE = 0.013, 95% CI: LL = 0.008 to UL = 0.019). This result indicated that severe FS increases PD, which in turn decreases PA, resulting in lower LS.

### Moderated mediation model: moderation for middle-aged and older

**Fig. 3:**
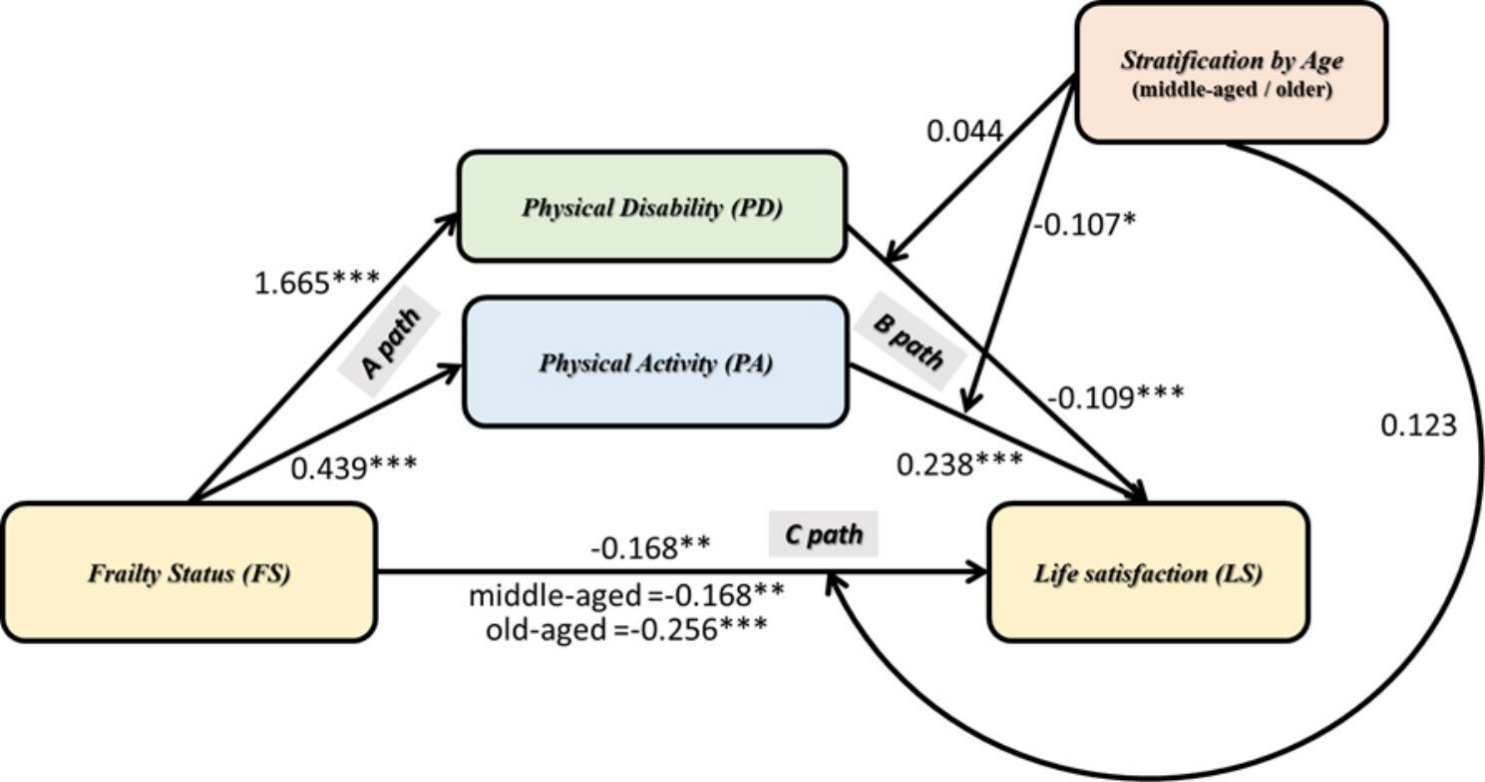
Moderated mediation model (n = 4,421) Conditional indirect effects on stratification by age (middle-aged coded as 0 and older as 1) of frailty status on life satisfaction through physical disability and physical activity. Standardised effects are presented. The moderated mediation model was adjusted for age, gender, and the number of chronic diseases. The effects on the direct path from FS to LS depict the conditional direct effects for each stratification by age as well as the unconditional direct effect on the C’ path (total effect C-path). The effects of the moderator’s stratification by age on the paths represent the interaction slopes. The effects on the B-paths from the mediators to LS represent the simple slopes. * p < 0.05, **P < 0.01, ***P < 0.001

A moderated mediation model was used to test whether the indirect effects of FS on LS through PD or PA are moderated by age stratification. The goal was to determine whether the previously observed mediation effect differed statistically significantly between subjects with middle-aged and older adults. The moderated mediation model was used to examine whether PD or PA acted as parallel mediators and whether stratification by middle and old age moderated the mediation. After adjustments for age, gender, and number of chronic diseases, the results showed statistically significant age-stratified interactions between FS (independent variable) and PD or PA (mediator variable), which were analyzed by simple linear regression. The moderation effect was observed on the mediator-dependent path (B path), and the direct path was independent of the dependent path (C path). The moderating effect was not observed in the independent variable to the mediating variable (A path). The slope of the indirect effects associated with the moderator variable was the index of moderated mediation (IMM). The statistical significance of the IMM effect was assessed along with the conditional indirect effects, which were assessed along with the stratification by middle-aged and older (shown in Fig. 3).

Through the moderated mediation model, we found that the interaction effect between FS and stratification by age had predictive power to affect LS (β = -0.087, SE = 0.077, p = 0.254). The stratification by age significantly moderated the mediational effect of PA (IMM = -0.107, SE = 0.052, p < 0.05) but not the mediational effect of PD (IMM = 0.044, SE = 0.032, p = 0.167). This indicated a meaningful difference in the magnitude of the conditional indirect effects of each stratification by age in the mediation effect of FS on LS through PA. Specifically, from the analysis of direct effects and indirect effects, it was found that the two direct effects and all indirect effects were significant without including zero. We observed that for PA for subjects who were middle-aged, the conditional indirect effects were strong and statistically significant (β = -0.105, SE = 0.018, LL = -0.140, UL = -0.070), and the same was true for subjects who were older (β = -0.058, SE = 0.015, LL = -0.088, UL = -0.028). Therefore, PA was a significant mediator in the relationship between FS and LS in subjects who were middle-aged and older. However, there was no meaningful difference in the mediating effect of PD on the relationship between FS and LS in subjects who were middle-aged and older.

## Discussion

People are ageing, not just as individuals or communities, but as a global population. While population ageing represents a triumph over disease, it also requires changing public health policy directions. Public health policy should not only focus on physiological directions but also ensure that psychological needs such as LS are met. The purpose of our study was to explore the co-occurrence and inter-relations between LS and physiological conditions such as FS, PD, and PA reported by middle-aged and older adults. We analyze the results related to physiological-related health problems in middle-aged and older adults with parallel mediation, serial mediation, and moderated mediation models to further clarify the research question.

### Geriatric frailty affects life satisfaction through physical disability and physical activity

Previous studies have confirmed that FS [35, 36], PD [37], and PA [38] in middle-aged and older adults have significant impacts on LS. In this study, we specifically aimed to demonstrate that FS, PD, and PA can form a common health problem cluster over the course of the ageing process. Our findings also showed that LS was negatively associated with FS and PD in middle-aged and older adults but positively associated with PA. This means that both middle-aged and older adults need to pay attention to these physiologically related health issues, with an emphasis on preventing frailty and focusing on PD and PA levels. Furthermore, parallel mediation analysis showed that PD and PA partially mediated the relationship between FS and LS. The results demonstrated the mediating effect of the physiological changes on FS and LS. This result can help provide a comprehensive direction for elderly care and have a positive impact on LS in the psychological dimension for middle-aged and older adults.

### The mediating variables of physical disability and physical activity have specific causal associations

The degrees of influence of ageing on life will depend on physiological changes and may also ignore the co-existence of physiological issues or the correlation between them. Much of the previous research has focused on strategies to reduce FS rather than management of the situations of physical incapacity or PA [39]. Although it is known that the advantages and disadvantages of physiological function can coexist with the severity of frailty [7], there are correlations with physiological states between PD and PA [40]. However, since there is no evidence of causal chains linked to interventions, health promotion programs may have imprecise designs in the future. The results of our study strongly indicate that, whether an individualised health promotion program is for middle-aged or older adults, it must be designed for the stratification of age. In addition, the content of the health promotion programs should not be limited to a single element. It should have multiple elements, including relieving PD and improving PA. A complete health promotion program can help improve the LS in frail middle-aged and older adults.

### The mediating effect between middle-aged and older adults

Preceding studies reported a correlation between PD and PA or similar variables that have been explored for middle-aged and older adults [41]. However, the results of our study are based on FS. Although our study was not longitudinal in design, it also confirms that the relationship between physiological-related health problems and psychological-related LS should be considered in both frailty care and public health policy directions. For example, when distinguishing the middle-aged and older strata, stratification can significantly reduce PA and have a mediating effect, but it does not have a mediating effect on PD. These new findings explain research conflicts in preceding studies and may resolve the understanding of variable interactions.

Before now, since the relationship between PD and PA has not been completely clear, systematic literature has pointed out that the combination of interventions such as physical training, nutritional supplements, cognitive training, health education, and home visits can improve the frailty of older people [42]. Past studies have suggested examining medications, social skills with nutrition [43], cognitive behavioral therapy with physical training [44], group exercise training, nutritional supplements, antidepressant medication or supportive psychotherapy, discontinuation of high-risk medications, and reduction of adverse home environments [45]. These composite interventions can effectively improve the PD of older people. In addition, the prevalence of insufficient physical activity has been found in more than half of middle-aged and older individuals. Their physical activity levels are lower than those recommended by WHO standards [11]. The extant research suggests that smart health care (eHealth) [46], PA monitors [47], or these measures in combination with telephone consultation [48] are effective in improving the PA status of older people. Overall, the combined interventions described above have shown efficacy in improving frailty, disability, and PA in older adults. Our study pointed out that FS will increase PD, which in turn affects PA, leading to a decrease in LS. This can be seen as evidence that the development of appropriate interventions in middle-aged and older adults can improve LS, and it is necessary to develop individualised compound intervention measures for the middle-aged or older, respectively. It is necessary to further explore whether a composite health promotion program can improve the FS of middle-aged and older adults, reduce PD, increase PA, improve LS, and strengthen the quality of elderly care.

### Limitations

This study included a large, nationally representative sample of participants and adjusted for potential confounding factors. However, education level and spousal status [49, 50], which are related to frailty factors and may affect the relationship between FS and PD, PA, and LS, were not assessed. However, our mediation analyses were adjusted for age [51], gender [52], and chronic diseases [53], which are key factors in LS and FS in older adults. Furthermore, the causality of the effect of FS on LS remains unclear due to the cross-sectional research design. The development of frailty and its effect on LS appear to progress slowly in middle-aged and older adults. The relationship between PD and PA may also change over time. The interlinked nature of variables prevents any assertion of causality or direction. The length of follow-up is important in following LS in middle-aged and older adults. A longer follow-up period would tend to weaken the strength of association for any variable that continues to be associated with the outcome. Future research should include longitudinal studies to clarify the physiological changes caused by time and their impact on LS in middle-aged and older adults.

## Conclusion

From the results of our study, FS, PD, and reduced PA often occur simultaneously in middle-aged and older adults. It provides evidence from two different samples of middle-aged or elderly populations. Our study also shows the direct and indirect effects of PD and PA on LS. The parallel mediation model showed that PD and PA can partially mediate the relationship between FS and LS in middle-aged and older adults. The serial mediation model showed that the severity of FS in causal chains is linked to increased PD, which in turn affects PA, leading to decreased LS to the extent that it mediates the relationship between FS and LS. The moderated mediation model revealed that different age stratifications can significantly moderate the mediating effect of PA but not that of PD. The findings of our study provide evidence for the assessment, prevention, and design of interventions for frailty, PD, and PA in middle-aged and older adults. Moreover, it is noted that PD and PA may have a causal relationship with the development of frailty. It may have a significant impact on LS in middle-aged and older adults.

## Electronic supplementary material

Below is the link to the electronic supplementary material.

Supplementary Material 1. Appendix 1. Pearson correlation coefficients between the variables (n = 4,421). Appendix 2. Models of the mediating role of physical disability and physical activity in the relationship between frailty status and life satisfaction (n = 4,421). Appendix 3. Standardised indirect effects for the paths on the SMMs. Appendix 4. Models of the moderation role of stratification by age (middle- and older) with a mediating role of physical disability and physical activity in the relationship between frailty status and life satisfaction (n = 4,421).  Appendix 5. direct and mediating effects on the different levels of stratification by age (n = 4,421).

## Data Availability

The data that support the findings of this study are available from Ministry of Health and Welfare of Taiwan, but restrictions apply to the availability of these data, which were used under license for the current study (Project No: R110025), and so are not publicly available. Data are however available from the corresponding author upon reasonable request and with permission from Taiwan’s Ministry of Health and Welfare.
