# Supplementary material for: The effect of research on life satisfaction in middle-aged and older adults: physical disability and physical activity as a parallel and serial mediation analysis
Source: BMC Geriatr. 2023 Mar 27;23:176. doi: 10.1186/s12877-023-03873-7 (PMC10044714; doi:10.1186/s12877-023-03873-7)
Supplement: Supplementary file 1 — Supplementary Material 1. Appendix 1. Pearson correlation coefficients between the variables (n = 4,421). Appendix 2. Models of the mediating role of physical disability and physical activity in the relationship between frailty status and life satisfaction (n = 4,421). Appendix 3. Standardised indirect effects for the paths on the SMMs. Appendix 4. Models of the moderation role of stratification by age (middle- and older) with a mediating role of physical disability and physical activity in the relationship between frailty status and life satisfaction (n = 4,421). Appendix 5. direct and mediating effects on the different levels of stratification by age (n = 4,421). [file 12877_2023_3873_MOESM1_ESM.docx]

**The effect of research on life satisfaction in middle-aged and older adults: Physical Disability and Physical Activity as a Parallel and Serial Mediation Analysis**

**Supplementary data**

- **Appendix 1.** Pearson correlation coefficients between the variables (*n* = 4, 421)

|  | FS | PD | PA | LS |
| --- | --- | --- | --- | --- |
| FS | 1 |  |  |  |
| PD | 0.689** | 1 |  |  |
| PA | -0.367** | -0.153** | 1 |  |
| LS | -0.248** | -0.215** | 0.182** | 1 |
| FS: frailty status; PD: physical disability; PA: physical activity; LS: life satisfaction  ** *p* < 0.01 | | | | |

- **Appendix 2.** Models of the mediating role of physical disability and physical activity in the relationship between frailty status and life satisfaction (*n* = 4, 421)

| Independent Variable | Mediator | Dependent Variables | Effect of X on M | Effect of M on Y | Direct Effect | Indirect Effect | Total Effect |
| --- | --- | --- | --- | --- | --- | --- | --- |
| FS | PD | LS | 1.665  (SE = 0.030)*** | -0.071  (SE = 0.013)*** | -0.221  (SE = 0.037)*** | -0.119  (-0.170 to -0.068) | -0.417  (SE = 0.026)*** |
|  | PA |  | -0.439  (SE = 0.015)*** | 0.176  (SE = 0.026)*** |  | -0.077  (-0.101 to -0.055) |  |
| FS: frailty status; PD: physical disability; PA: physical activity; LS: life satisfaction  adjusted age, gender and chronic disease  ** *p* < 0.01；****p* < 0.001 | | | | | | | |

- **Appendix 3.** Standardised indirect effects for the paths on the SMMs

| Serial mediation model | The indirect effects of frailty on LS | |
| --- | --- | --- |
|  | *b* (boot SE) | BC 95% CI (LL-UL) |
| SMM1: FS → PD → LTPA → LS |  |  |
| A. FS → PD → LS | -0.119 (0.026) | -0.170 to -0.069 |
| B. FS → PA → LS | -0.090 (0.013) | -0.116 to -0.065 |
| C. FS → PD → PA → LS | 0.013 (0.003) | 0.008 to 0.019 |
| SMM2: FS → PA → PD → LS |  |  |
| A. FS → PA → LS | -0.077 (0.012) | -0.101 to -0.054 |
| B. FS → PD → LS | -0.124 (0.028) | -0.178 to -0.070 |
| C. FS → PA → PD → LS | 0.005 (0.002) | 0.002 to 0.008 |
| Table shows standardised indirect effects with bootstrapped SEs.  ∫Bias-corrected 95% CIs.  *LL: Lower Limit; UL: Upper Limit  SMM: Serial mediation model; FS: Frailty status; PA: physical activity; PD: physical disability; LS: life satisfaction | | |

- **Appendix 4.** Models of the moderation role of stratification by age (middle- and older) with a mediating role of physical disability and physical activity in the relationship between frailty status and life satisfaction (*n* = 4, 421)

| Dependent Variables | Predictor variable | Effect | |
| --- | --- | --- | --- |
| LS | FS | -0.168 | (SE = 0.062)** |
|  | PD | -0.109 | (SE = 0.029)*** |
|  | PA | 0.238 | (SE = 0.040)*** |
|  | SA | 0.123 | (SE = 0.146) |
|  | FS x SA | -0.087 | (SE = 0.077) |
|  | PD x SA | 0.044 | (SE = 0.032) |
|  | PA x SA | -0.107 | (SE = 0.052)* |
| PA: physical activity; PD: physical disability; LS: life satisfaction; SA: Stratification by Age  adjusted age, gender and chronic disease  * *p* < 0.05；** *p* < 0.01；****p* < 0.001 | | | |

- **Appendix 5.** direct and mediating effects on the different levels of stratification by age (*n* = 4, 421)

| Antecedent variable | | Direct Effect | Indirect Effect | |
| --- | --- | --- | --- | --- |
|  |  |  | PD | PA |
| moderation | middle-aged | -0.168  (SE = 0.062)**  (-0.289 to -0.048) | -0.181  (SE = 0.060)  (-0.301 to -0.068) | -0.105  (SE = 0.018)  (-0.140 to -0.070) |
|  | older | -0.256  (SE = 0.046)***  (-0.345 to -0.167) | -0.107  (SE = 0.030)  (-0.167 to -0.051) | -0.0578  (SE = 0.015)  (-0.088 to -0.028) |
| PA: physical activity；PD: physical disability  adjusted age, gender and chronic disease  ** *p* < 0.01；****p* < 0.001 | | | | |
